# Detection of cognitive decline by spinal posture assessment in health exams of the general older population

**DOI:** 10.1038/s41598-022-12605-7

**Published:** 2022-05-19

**Authors:** Hikaru Nishimura, Shota Ikegami, Masashi Uehara, Jun Takahashi, Ryosuke Tokida, Hiroyuki Kato

**Affiliations:** 1 Rehabilitation Center, Shinshu University Hospital, 3-1-1 Asahi, Matsumoto, Nagano 390-8621 Japan; 2 Department of Orthopaedic Surgery, Shinshu University School of Medicine, 3-1-1 Asahi, Matsumoto, Nagano 390-8621 Japan

**Keywords:** Public health, Neurological disorders, Epidemiology, Orthopaedics

## Abstract

The recent increase in the older adult population has led to a higher prevalence of cognitive impairment, which is often overlooked in routine health examinations. Citizens aged 50–89 years were targeted for this cohort survey by random sampling from the resident registry of a cooperating town in 2014. A total of 411 participants (202 male and 209 female) were enrolled. We analyzed the distribution of cognitive function test scores as determined by Montreal Cognitive Assessment and Mini-Mental State Examination tests in each age (50’s, 60’s, 70’s and 80’s) and sex group to examine whether cognitive decline could be detected by sagittal spinal balance measurement based on a radiological approach. Sagittal spinal balance was quantitatively measured as sagittal vertical axis (SVA). We observed significant associations for higher age and/or SVA anteriorization with lower cognitive function. In males, spinal balance anteriorization was associated with cognitive decline independently of age, with combinations of age and SVA also making valid cognitive decline determinations; male cases of SVA ≥ 100 mm at any age, SVA ≥ 90 mm at ≥ 70 years, and SVA ≥ 70 mm at ≥ 80 years were all more likely to have cognitive decline than cases below those values. For females, cognitive decline was more likely in cases of SVA ≥ 70 mm, regardless of age. Thus, spinal balance anteriorization can be regarded as an easily visible indicator of latent cognitive decline in community-dwelling older people.

## Introduction

The recent rise in disability rates among older people has become increasingly alarming in consideration of caregiver costs and associated impairments in quality of life (QOL)^1^. As cognitive function diminishes with age, mild cognitive impairment (MCI) is a transitional condition between cognitive decline during the normal aging process and very early dementia. Although the definition of MCI continues to change, the current consensus includes self- or informant-reported cognitive complaints, objective cognitive impairment, preserved independence in functional abilities, and no dementia^2–5^. MCI often causes problems with memory, language, thinking, and judgment but does not comprise dementia; thus, some cases of MCI do not deteriorate further. In individuals over the age of 70 years, 14% have sufficient cognitive impairment to warrant a diagnosis of dementia^6^. Gait slowing is also common among patients with this condition^7–9^. MCI rarely has a critical impact on daily life but is nonetheless a pre-disease condition with a very high probability of progressing to dementia. Several reports have shown that activities of daily living (ADL) and instrumental ADL (IADL) are already adversely affected at the MCI stage^10,11^.

It is widely recognized that sagittal spinal balance deteriorates with age^12,13^, which may hinder standing and walking in advanced cases^14^. Sagittal spinal alignment correlates strongly with health-related QOL, with severe spinal imbalance known to impair QOL results^15^. Indeed, sagittal spinal balance is an important barometer of health status in seniors. Whereas severe spinal imbalances accompanied by changes in spinal alignment are now recognized as adult spinal deformity (ASD), minor or moderate changes in alignment are often considered a natural process of aging.

Although spinal alignment change and cognitive deterioration are individually acknowledged as age-related phenomena, no studies have examined for associations between those factors. Various events of age-related decline occur simultaneously or in tandem, with each not completely independent of the others. This concept has been introduced using the term "frailty" as a way of understanding the changes in health in older people^16^. We maintain that spinal imbalance is not just a problem in itself, but rather one of the manifestations of several potential frailties in older adults.

For the construction of a new population study of Japanese community-dwelling older people, eligible candidates were randomly sampled from the basic resident registry of a cooperating town in an inland rural area to minimize selection bias and obtain a cohort more representative of the general population. This epidemiological study of comprehensive locomotive health in older people was coined “the Obuse study”, bearing the name of the collaborating local government.

The hypothesis of the current study is that diminished cognitive function is detectable by the degree of spinal balance anteriorization. As such, this investigation aimed to determine whether cognitive deterioration could be detected by spinal posture assessment in the general older population using the Obuse study cohort.

## Results

**Figure 1:**
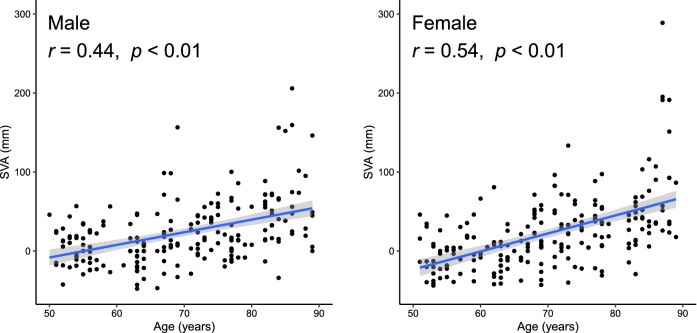
Distributions of sagittal vertical axis with age. *r* denotes Pearson’s correlation coefficient between sagittal vertical axis and age. *SVA* sagittal vertical axis.

Among the participants, the mean ± standard deviation distribution of male and female sagittal vertical axis (SVA) measurements was 22 ± 41 mm and 22 ± 46 mm, respectively. The SVA values for each age group are shown in Table 1. For both sexes, a moderate positive correlation was seen for age and SVA (Fig. 1). Larger SVAs of ≥ 50 mm were more frequent in older age groups.

**Table 1:** Spinal alignment parameter findings, cognitive function test scores, and prevalence rates of cognitive impairment.

Sex	Age (years)	N	SVA (mm)	MoCA		MMSE	
				Score	< 26 points	Score	< 24 points
Male	50's	50	6 ± 26	27 ± 2	14 (28%)	29 ± 1	0 (0%)
	60's	53	9 ± 38	26 ± 3	22 (42%)	28 ± 2	1 (2%)
	70's	55	22 ± 30	24 ± 3	35 (64%)	27 ± 2	4 (7%)
	80's	44	56 ± 49	21 ± 4	38 (86%)	26 ± 3	7 (16%)
Female	50's	46	− 5 ± 27	27 ± 2	7 (15%)	29 ± 1	0 (0%)
	60's	61	5 ± 30	26 ± 3	25 (41%)	28 ± 2	2 (3%)
	70's	54	30 ± 36	25 ± 3	29 (54%)	28 ± 2	2 (4%)
	80's	48	61 ± 60	21 ± 4	39 (81%)	26 ± 2	5 (10%)

Values represent the mean ± standard deviation or number of cases concerned (rate). The maximum MoCA score is 30 points, with a score of ≥ 26 considered normal (i.e., without cognitive decline). The maximum MMSE score is 30 points, with a score of ≥ 24 regarded as normal (i.e., without suspected dementia).

*SVA* sagittal vertical axis, *MoCA* montreal cognitive assessment, *MMSE* mini-mental state examination.

SVA was significantly associated with physical QOL scores. The Physical Component Summary (PCS) score of the SF-8 Health Survey was evaluated in groups of SVA < 50 mm (balanced), SVA 50–100 mm (mildly anteriorized), and SVA > 100 mm (severely anteriorized). In males, the mean ± standard deviation PCS scores were 49 ± 7, 44 ± 9, and 41 ± 10 in the balanced, mildly anteriorized, and severely anteriorized groups, respectively. These scores were 49 ± 6, 42 ± 8, and 36 ± 11, respectively, in females. In males, PCS scores were significantly lower in the mildly and severely anteriorized groups than in the balanced group (*p* < 0.01 and *p* = 0.01 for balanced vs. mildly anteriorized and balanced vs. severely anteriorized, respectively, Tukey–Kramer multi-comparison method). In females, a significant difference was detected among all groups (*p* < 0.01, *p* < 0.01, and *p* = 0.01 for balanced vs. mildly anteriorized, balanced vs. severely anteriorized, and mildly anteriorized vs. severely anteriorized, respectively). Larger SVA values tended to display lower PCS scores. On the other hand, no significant relationships were observed between SVA and SF-8 Mental Component Summary (MCS) scores.

Cognitive function test scores tended to decrease with age for both genders (Table 1). Mean Montreal Cognitive Assessment (MoCA) scores were the normal limit of 26 points in males and females in their 60's, with cognitive decline indicated in the 70's and 80's age groups. The prevalence rate of cognitive decline in males and females in their 50's was 28% and 15%, respectively, which increased by decade to more than half of participants in their 70's and over 80% of participants in their 80's. The individual testing methods did not differ significantly between genders (mean score: 25 points for both sexes for MoCA [*p* = 0.47] and 28 points for both sexes for Mini-Mental State Examination [MMSE] [*p* = 0.89]).

Table 2 demonstrates how age and SVA are associated with cognitive decline based on MoCA score, with additional analysis on the relationship between cognitive decline and worsening spinal alignment and links to frailty. In males, older age and greater SVA were both significantly and independently associated with cognitive decline. In univariate analysis, employment in the tertiary sector of economy was associated with decreased cognitive decline, although significance was lost in multivariate analysis. In females, age, SVA, osteoporosis, and subjective fatigue were associated with cognitive decline in univariate analysis, with a negative association for employment in the tertiary sector of economy. In multivariate analysis, age alone remained significantly associated with cognitive decline.

**Table 2:** Univariate and multivariate analyses on the association of cognitive impairment with age, spinal balance, and other candidate factors.

Sex	Candidate	Univariate analysis		Multivariate analysis	
		Odds ratio	*p*-value	Odds ratio	*p*-value
Male	Age (+ 10 years)	2.5 (1.9–3.4)	< 0.01*	1.8 (1.2–2.7)	< 0.01*
	SVA (+ 10 mm)	1.3 (1.1–1.4)	< 0.01*	1.2 (1.01–1.3)	< 0.01*
	BMI (+ 1 kg/m^2^)	1.0 (0.93–1.1)	0.76	2.4 (1.7–3.4)	< 0.01
	Osteoporosis (+)	0.56 (0.09–3.4)	0.53		
	Spine disease (+)	0.2 (0.02–1.9)	0.16		
	Arthritis (+)	1.0 (0.53–2.0)	0.93		
	Low back pain (VAS + 10 mm)	1.1 (0.99–1.3)	0.08	1.1 (0.96–1.3)	0.15
	Weight loss within 6 months (+)	1.8 (0.88–3.8)	0.10		
	Subjective fatigue (+)	1.4 (0.65–3.0)	0.40		
	**Job (vs. unemployed/ retired)**				
	Primary sector	0.84 (0.38–1.9)	0.66	1.2 (0.50–2.8)	0.68
	Secondary sector	0.40 (0.12–1.3)	0.13	1.7 (0.41–6.8)	0.47
	Tertiary sector	0.16 (0.07–0.35)	< 0.01*	0.49 (0.18–1.3)	0.16
Female	Age (+ 10 years)	2.7 (2.0–3.7)	< 0.01*	2.5 (1.7–3.7)	< 0.01*
	SVA (+ 10 mm)	1.2 (1.1–1.3)	< 0.01*	1.0 (0.94–1.1)	0.53
	BMI (+ 1 kg/m^2^)	1.0 (0.93–1.1)	0.82		
	Osteoporosis (+)	3.0 (1.3–6.8)	0.01*	0.8 (0.28–2.1)	0.61
	Spine disease (+)	0.0 (0.0-Inf)	0.98		
	Arthritis (+)	1.1 (0.61–2.1)	0.70		
	Low back pain (VAS + 10 mm)	1.1 (0.95–1.2)	0.29		
	Weight loss within 6 months (+)	2.6 (0.94–7.0)	0.06	2.5 (0.77–7.8)	0.13
	Subjective fatigue (+)	2.5 (1.2–5.1)	0.01*	1.8 (0.75–4.2)	0.19
	**Job (vs. unemployed/ retired)**				
	Primary sector	0.96 (0.48–1.9)	0.89	1.3 (0.58–2.8)	0.55
	Secondary sector	0.79 (0.24–2.7)	0.70	1.9 (0.49–7.5)	0.34
	Tertiary sector	0.33 (0.16–0.67)	< 0.01*	0.77 (0.33–1.8)	0.55

Odds ratios are presented with the 95% confidence interval.

*SVA* sagittal vertical axis, *BMI* body mass index, *VAS* visual analogue scale, *Inf* infinite.

*p < 0.05.

**Figure 2:**
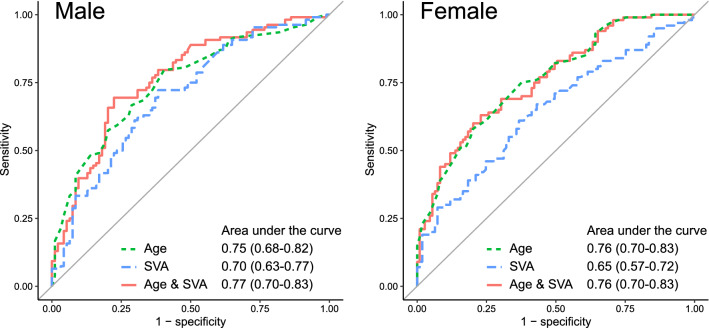
Receiver operating characteristic curves of cognitive decline prediction based on age and/or sagittal vertical axis. *SVA* sagittal vertical axis.

Figure 2 presents the receiver operating characteristic (ROC) curves of cognitive decline prediction based on age and SVA scores. For males, the area under the ROC curve (AUC) for age alone, SVA alone, and the combination of age and SVA all exceeded 0.7, indicating relatively good cognitive decline prediction. In particular, the predictive ability of combined age and SVA was high, with the lower limit of the 95% confidence interval of the AUC exceeding 0.7. For females, the AUC for age, but not SVA was over 0.7 for cognitive decline prediction. Combining age and SVA did not raise the predictive value above that of age alone.

Table 3 shows the positive likelihood ratios for cognitive decline determination for each cutoff combination of age and SVA. For males, several combinations could make valid cognitive decline determinations. Cases with SVA ≥ 100 mm at any age, SVA ≥ 90 mm at ≥ 70 years, and SVA ≥ 70 mm at ≥ 80 years were all more likely to have cognitive decline than those below those values. Females with SVA ≥ 70 mm were also more likely to have cognitive decline, regardless of age.

**Table 3:** Positive likelihood ratio matrix for mild cognitive impairment prediction.

Sex	Age (years)	SVA (mm)						
		Any SVA	≥ 50	≥ 60	≥ 70	≥ 80	≥ 90	≥ 100
Male	Any age	–	3.37	2.74	2.79	1.96	2.32	6.09^†^
	≥ 60	1.41	3.16	2.74	2.79	1.96	2.32	6.09^†^
	≥ 70	2.32	3.77	3.30	4.06	3.48	6.09^†^	5.22^†^
	≥ 80	4.60	3.92	4.06	5.22^†^	6.09^†^	Inf^††^	Inf^††^
Female	Any age	–	2.73	3.43	5.18^†^	9.27^†^	6.54^†^	8.72^†^
	≥ 60	1.45	2.73	3.43	5.18^†^	9.27^†^	6.54^†^	8.72^†^
	≥ 70	2.18	3.27	3.63	6.54^†^	8.72^†^	6.54^†^	8.72^†^
	≥ 80	4.72	4.63	4.72	13.08^††^	11.99^††^	10.90^††^	7.63^†^

Values indicate positive likelihood ratios for the combination of age and SVA. Ratios ≥ 5^†^ and ≥ 10^††^ are indicated.

*SVA* sagittal vertical axis, *Inf* infinite.

## Discussion

In the present cohort study of over 400 participants randomly selected from a rural Japanese town registry, the prevalence rate of cognitive decline reached more than half among participants in their 70's and over 80% among those in their 80's. We observed that cognitive decline could be reliably detected by combining age and the degree of spinal imbalance. Males with SVA ≥ 100 mm at any age, SVA ≥ 90 mm at ≥ 70 years, and SVA ≥ 70 mm at ≥ 80 years were all likely to have cognitive decline. Females with SVA ≥ 70 mm were also likely to have cognitive decline irrespectively of age. The combination of age and spinal balance were able to detect latent cognitive decline based on five–tenfold likelihood contrasts. In contrast, it could not rule out cognitive decline at any age and spinal balance, nor could it reveal any value as a gatekeeper measurement.

Impaired walking, standing, and standing balance have been related to elevated SVA^17^, and previous studies showed SVA to be correlated with balance ability^18,19^. Diminished walking, standing, and balance have been associated with poor cognitive function in older people as well^20^. Therefore, we hypothesized that spinal imbalance might not only indicate poor motor function, but also reduced cognitive ability.

ASD and frailty are closely and interactively related^21^. Patients with ASD cannot exercise muscular strength sufficiently and often exhibit symptoms of intermittent claudication due to back pain^22^. Pressure on the abdomen may also cause losses in appetite and weight and contribute to frailty. Frailty is a pre-disease condition comprehensively summarizing the symptoms accompanying aging and includes the concept of a decline in social activity due to mental and physical changes. Although frailty is reversible, the patient’s ASD may deteriorate permanently in severe cases.

On the other hand, spinal alignment changes less severe than those in ASD are not included in frailty. Such alterations tend to be regarded as a natural consequence of age. However, cognitive decline with spinal alignment changes may be a factor contributing to frailty through mental and social decline. On report found an association between severe kyphosis patients and diminished health-related QOL^23^. Although it remains difficult to prove a direct causal relationship between postural changes and cognitive decline, our results demonstrate that spinal anteriorization and cognitive decline are correlated phenomena occurring simultaneously with age. Thus, when visible appearance changes in posture occur, appropriate diagnostic measures are advised in consideration of the possibility of cognitive impairment to help prevent frailty, dementia, and bedridden status.

Considerable research has been conducted on the relationship between motor and cognitive function, including the Obuse study^20^. When muscle weakness, decreased walking ability, and other forms of physical function deterioration are present, a risk of AD and dementia may exist^24–27^. In addition, cognitive function deterioration was identified as a risk factor for physical function decline^28^. In the broad sense of the term, cognitive frailty is a condition in which physical and cognitive decline coexist. Cognitive frailty is a high-risk disorder that has gained attention in recent years^29^. Nara et al. described that a MoCA cut-off score of 23 might be useful to predict cognitive function at a potentially reversible stage with exercise training in community-dwelling Japanese older adults with cognitive decline^30^. Of the 411 subjects in the present study, there were 119 (29%) who scored < 26 points and ≥ 23 points for MoCA. If cognitive decline can improve with appropriate intervention in this population, it may be desirable to adopt a variety of approaches to such a condition. The mild deterioration of cognitive function is a difficult parameter to notice among the age-related changes that lead to frailty. Just as osteoporosis has been under-detected in the older population, cognitive deterioration may suffer from this problem as well. Testing that requires special equipment or additional time, even that with good detection capability, may be limited as an exam method in the older population. We herein propose a method to detect cognitive decline based on SVA, a representative radiological indicator of spinal balance anteriorization that may incidentally be obtained in other examinations. However, a more intuitive method for noticing spinal posture changes may be more efficient in revealing latent cognitive decline.

The limitations of the current investigation include a cross-sectional design and the possibility of selection bias. First, since this was a cross-sectional study, the causal relationship between sagittal spinal balance and cognitive function could not be established. Cognitive function is influenced by numerous direct factors which include genetic, health, and environmental sources. Due to the multifactorial nature of cognitive function, the present study investigating indirect factor (i.e., sagittal spinal alignment) associations should have ideally considered more possible confounders. However, it was not possible to completely account for the myriad of relevant factors, especially potential ones, and longitudinal studies using this investigation as a prelude are being planned. Furthermore, sagittal spinal alignment is also considerably influenced by numerous factors, such as age, lifestyle, disability of physical activities, osteoporosis, degenerative joint disease, spinal disease including lumbar stenosis and degenerative sagittal imbalance, and pain; even in the same person, sagittal spinal alignment may change daily according to his or her physical condition. As this study merely presents correlations, longitudinal research is necessary to exclude the effects of relevant confounders and address the basis of the association, including causal relationships. Longitudinal studies will also help reveal which of spinal balance anteriorization or cognitive decline occurs first. Second, since this was a non-compulsory survey, the proportion of randomly sampled people who ultimately participated in the survey was less than half, implying incomplete selection bias elimination. Nevertheless, the Obuse study cohort closely resembled the average Japanese population due to its distinctive survey design. In order to maintain ADL, IADL, and QOL in older people, it will be necessary to monitor for signs of impending cognitive impairment that may lead to frailty and dementia. Our results showed that the anteriorization of spinal balance existed at the onset of cognitive impairment. Accordingly, greater attention is warranted to changes in posture among community-dwelling older people. Lastly, potential human error in the measurement of radiographic angles is an important limitation that must be mentioned. Advanced evaluation techniques, such as the use of machine learning, are being considered to improve the accuracy and reliability of measurements for future work.

In conclusion, spinal balance anteriorization was significantly associated with cognitive function decline in Japanese older adults. Cognitive decline could be reliably detected by combining age and the degree of spinal imbalance. Males with SVA ≥ 100 mm at any age, SVA ≥ 90 mm at ≥ 70 years, and SVA ≥ 70 mm at ≥ 80 years are likely to have cognitive decline, while females with SVA ≥ 70 mm at any age are likely to harbor this condition. Such visible clues as the anteriorization of spinal balance can help to more easily monitor for signs of impending cognitive impairment, which may lead to dementia and frailty.

## Methods

This study was approved by the investigational review board of Shinshu University Hospital (approval number: 2792) and performed in accordance with the Declaration of Helsinki and the STROBE Statement. All participants provided written informed consent for study participation.

### Construction of cohort classified by sex and age group

The participant group in this study was the Obuse study cohort based on a basic municipal resident registry. The construction procedure of the cohort is described in a previous report^31^. Briefly, we randomly sampled 1,297 individuals from 5352 people aged between 50 and 89 years in the basic resident registry of Obuse town in 2014. After providing written consent, 415 participants were enrolled in the Obuse study. A total of 411 participants (202 male and 209 female) were included after the exclusion of 4 candidates, which included 2 candidates unable to participate in radiological assessments, 1 candidate already diagnosed as having dementia, and 1 candidate with a postural disorder due to Parkinson's disease. Table 4 summarizes the characteristics of each group. The primary employment sector was related to the agricultural industry, the secondary sector was manufacturing, and the tertiary sector was the service industry. The regional characteristics of the cooperating town included rural and urban areas, with high levels of employment in the agricultural and service industries. Thus, the participants were mainly employed in the primary and tertiary sectors, with relatively few in the secondary sector. The proportion of unemployment increased with age, especially since the general retirement age in Japan is 60–65 years old.

**Table 4:** Characteristics of the study cohort.

Sex	Age group (years)	N	Age (years)	Height (cm)	Weight (kg)	BMI (kg/m^2^)	Job (Pri; Sec; Ter; None)
Male	50's	50	54.5 ± 2.3	171.8 ± 6.0	67.1 ± 9.1	22.7 ± 2.9	3; 7; 40; 0
	60's	53	65.2 ± 2.5	166.7 ± 4.7	66.9 ± 7.7	24.1 ± 2.7	18; 5; 19; 11
	70's	55	74.9 ± 2.4	163.2 ± 5.0	60.0 ± 10.3	22.5 ± 3.4	22; 2; 8; 23
	80's	44	84.6 ± 2.6	160.2 ± 5.7	57.6 ± 8.5	22.4 ± 2.8	19; 0; 3; 22
	All	202	69.4 ± 11.1	165.6 ± 6.8	63.1 ± 9.8	23.0 ± 3.0	62; 14; 70; 56
Female	50's	46	55.0 ± 2.5	158.1 ± 5.0	55.4 ± 9.1	22.2 ± 3.8	5; 4; 29; 8
	60's	61	65.4 ± 3.0	152.8 ± 5.4	52.2 ± 7.6	22.3 ± 2.8	21; 4; 17; 19
	70's	54	74.7 ± 2.6	149.7 ± 5.3	50.6 ± 7.9	22.6 ± 3.2	16; 4; 8; 26
	80's	48	85.0 ± 2.2	144.6 ± 5.9	48.3 ± 7.9	23.1 ± 3.3	11; 0; 5; 32
	All	209	70.0 ± 11.0	151.3 ± 7.1	51.6 ± 8.4	22.5 ± 3.3	53; 12; 59; 85

Values represent the mean ± standard deviation.

*BMI* body mass index, *Pri* primary sector, *Sec* secondary sector, *Ter* tertiary sector.

### Measurements of spinal alignment

**Figure 3:**
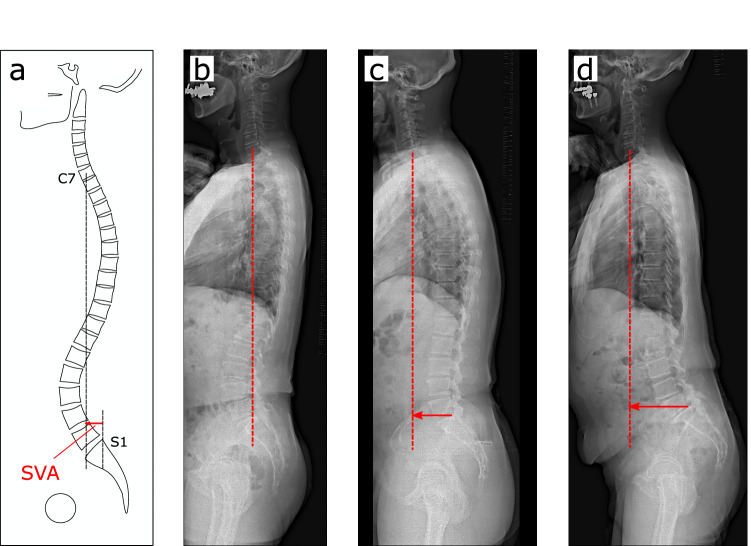
Sagittal vertical axis as a radiological parameter representing sagittal spinal balance. (**a**) SVA is the distance from the plumb line from the center of C7 to the posterior edge of the upper sacral endplate surface; (**b**) SVA = 0 mm, which is in sagittal balance; (**c**) SVA = 70 mm, which represents sagittal balance anteriorization; (**d**) SVA = 95 mm, which is highly anteriorized and indicates sagittal imbalance. *SVA* sagittal vertical axis.

All participants underwent whole-spine lateral radiography in a standing position (fists on clavicles position) for the measurement of SVA as a parameter of sub-cervical total spinal alignment (Fig. 3). SVA was measured as the distance from the plumb line from the center of C7 to the posterior edge of the upper sacral endplate surface. The averaged values of 2 spine surgeons (M.U. and S.I.) and a trained staff member (R.T. or H.N.) were calculated for the analysis. Inter-rater reliability was high at 0.95.

### Cognitive function testing

We employed 2 tests to evaluate cognitive function: MoCA and MMSE. As a tool to determine the existence of MCI, the 30-point MoCA provides higher a sensitivity and specificity than does the 30-point MMSE, another representative cognitive function evaluation method^32,33^. Participants with ≥ 26 points for MoCA were considered normal (sensitivity: ≥ 94%, specificity: ≤ 60%)^34^, with those scoring ≤ 25 points as having cognitive impairment^32^. Similarly for MMSE, a score of < 24 points was judged as suspected dementia^35,36^. The Japanese version of both questionnaires was used. The cognitive impairment measured in this epidemiological study was defined solely by screening test (MoCA and MMSE) scores. For analysis, we regarded the presence of cognitive decline as a MoCA score of < 26 points. In order to precisely define cognitive impairment, including MCI, as a disease requiring treatment, however, further comprehensive examination and exclusion of other conditions are required^37^.

### Health-related QOL assessments and other interview information

SF-8 Health Survey measures were determined for all participants for health-related QOL evaluation. Results were calculated and expressed as 2 summary scores: PCS and MCS. Information related to frailty was also collected via interviews, including that on osteoporosis, spine and joint disease, weight loss within 6 months, subjective fatigue, and degree of low back pain (visual analogue scale of 0–100 mm).

### Statistical analyses

The relationship between age and SVA was assessed by Pearson’s correlation coefficient for each gender. The mean and standard deviation of the cognitive function test scores for each age and sex group were calculated along with the prevalence of cognitive impairment. Calculated cognitive function testing results between genders were assessed using Welch’s *t*-test. In the evaluation of the impact of age and SVA on cognitive decline, we employed univariate and multivariate logistic analyses with the presence of cognitive decline as the response variable and age and SVA as the explanatory variables. In addition to age and spinal alignment, BMI and information obtained from interviews (history of osteoporosis, spinal disease, arthritis, degree of low back pain, weight loss within 6 months, subjective fatigue, and job category) were included in the analyses as candidate factors. Candidate factors with *p*-values of < 0.1 in univariate analysis were included in subsequent multivariate analysis.

ROC analysis was performed to assess the power of cognitive decline detection by age and/or SVA. In AUC calculations, a value of ≥ 0.7 was considered a sufficient curve pattern for reliable prediction. Afterwards, matrices of positive likelihood ratios were constructed for combinations of age and SVA, whereby a positive likelihood ratio of ≥ 5.0 was considered useful for a suspected diagnosis and a ratio of ≥ 10.0 being considered a confident diagnosis. Positive likelihood ratios of < 5.0 were interpreted as having no screening value.

Statistical analyses were carried out using the statistical package R, version 3.6.2 (available at: http://www.r-project.org). The level of significance was set at *p* < 0.05.
